# Supplementary material for: Insights into the Resistome and Phylogenomics of a ST195 Multidrug-Resistant Acinetobacter baumannii Clinical Isolate from the Czech Republic
Source: Life (Basel). 2021 Oct 13;11(10):1079. doi: 10.3390/life11101079 (PMC8537504; doi:10.3390/life11101079)
Supplement: Supplementary file 1 [file life-11-01079-s001.zip › life-1268491-SI.pdf]

life

Supplementary

Table S1. Primers used to detect the β-lactamase genes using PCR.

Primer Name	Sequence (5' to 3' direction)[a]	Target	Amplicon Size (bp)	Tm (⁰C)	Reference
ADC-F1/R1	MAACCTAAAAACYCAATCGGTG, YGGATAAGMAAACTCTTCCCA	ADC	417	58	[1]
ADC-F2/R2	RGGTTTCTAYCAAGTCGGYA, GCGTTCTTCATTBGGAATACGT		268	59	[1]
CARB-F/R	GGGAAAACGTTGGGAACAT, TAATAGCACGCGACCCATA	CARB	578	54	[1]
CTX-F1/R1	ATGTGCAGYACCAGTAARGT, TGGGTRAARTARGTSACCAGA	CTX	593	55	[2]
CTX-F2/R2	ATGTGCAGYACCAGYAAAG, GGCCARATCACCGCRATAT		551	56	[1]
GES-F/R	ACGTTCAAGTTTCCGCTAG, GGCAACTAATTCGTCACGT	GES	624	53	[3]
IMP-F/R	GAGTGGCTTAATTCTCRATC, CCAAACYACTASGTTATCT	IMP	183	50	[4]
KPC-F/R	CGCTAAACTCGAACAGGAC, CGGTCGTGTTTCCCTTTAG	KPC	548	54	[1]
NDM-F/R	GGGGATTGCGACTTATGC, AGATTGCCGAGCGACTTG	NDM	258	53	[5]
OXA(1)-F/R	ATTTCAAGCCAAAGGCACGA, GCCACTCAACCCATCCTACC	OXA-2-like	569	57	[6]
OXA(2)-F/R	ACTTGCTATGTGGTTGCTTCTC, ACCTTTTCTCGCCCTTCCAT	OXA-23-like	310	56	[5]
OXA(3)-F/R	GCAGAAAGAAGTAAAGCGGG, CCAACCAGTCAACCAACCT	OXA-24-like	271	54	[5]
OXA(4)-F/R	AACGGGCGAACCAAGCATTTT, TGAGCACTTCTTTTGTGATGGCT	OXA-48-like	597	57	[6]
OXA(5)-F/R	TGTACCTGCTTCGACCTTCA, TCCCCAACCACTTTTTGCGT	OXA-51-like	436	54	[6]
OXA(6)-F/R	TAGACCGAGCAAAAACAGC, CAATTGCCCTTGGGCTAAA	OXA-58-like	365	52	[7]
OXA(7)-F/R	CCTCTCAAATCACTTCAACTCAATCT, GCTGCAAAACTGCGCTTCTT	OXA-134-like	253	58	[5]
OXA(8)-F/R	ACGAGCACATACAGAATATGTCCC, CCATTAGATTTTTCTACCCAACCTGT	OXA-143-like	510	59	[5]
OXA(9)-F/R	CTACGGCAATGACTTGAGCC, CCTGATAAACGGGAACGGCA	OXA-211-like	209	57	[7]
OXA(10)-F/R	ATRTTCCCGCCTCTACCTTT, YATCCCATCCCCAACCRCTT	OXA-213-like	444	56	[7]
OXA(11)-F/R	GCGTGAAGAAGTGAAGCGTGT, TCAACATCCATTCCCCAACCAC	OXA-228-like	243	57	[7]
PER-F/R	TACCTGGGCTCCGATAATG, TTCGACCATCCACTTCCAT	PER	336	54	This study
SHV-F/R	AACAGCYGGAGCGAAAGAT, CYTATCGGCGATAAACCAGC	SHV	437	55	This study
TEM-F/R	CACCAGTCACAGAAAAGCA,	TEM	450	54	[1]

VEB-F/R	AGGGCTTACCATCTGGC TTTCCGATTGCTTTAGCCG, CCCCAACATCATTAGTGGC	VEB	553	54	[1]
VIM-F/R	CGCGGAGATTGARAAGCAAA, CGCAGCACCRGGATAGAARA	VIM	247	57	[8]

[a]For degenerate primers: B = C or G or T; M = A or C; R = A or G; S = G or C; Y = C or T.

Table S2. Distribution of locally colinear blocks (LCBs) of *Acinetobacter baumannii* AC30 relative to strain 11069/A.

LCB	Size (bp)	Coordinates*	GC (%)
1	167,750	3 - 167,752	40.2
2	38,738	167,765 - 206,502	37.3
3	218,443	206,514 - 424,956	38.6
4	27,837	424,965- 452,801	38.4
5	93,500	452,806 - 546,305	39.9
6	140,237	546,310 - 686,545	37.8
7	25,878	686,553 - 712,430	39.2
8	404,514	712,435 - 1,115,603	38.7
9	2,853	1,115,628 - 1,118,480	37.0
10	233,136	1,118,500 - 1,350,917	38.5
11	8,841	1,359,767 - 1,350,927	40.0
12	1,965	1,359,772 - 1,361,736	39.1
13	119,136	1,361,741 - 1,479,923	38.8
14	117,986	1,479,927 - 1,597,906	38.3
15	81,000	1,660,427 - 1,660,507	35.2
16	2,035	1,668,542 - 1,670,576	28.4
17	29,153	1,700,868 - 1,671,716	40.4
18	51,856	1,700,872 - 1,752,066	39.5
19	128,561	1,752,164 - 1,880,724	40.5
20	27,050	1,881,358 - 1,908,407	39.4
21	115,052	1,908,411 - 2,023,462	39.1
22	596,641	2,023,466 - 2,612,935	39.2
23	15,678	2,686,770 - 2,702,447	39.9
24	18,564	2,702,452 - 2,721,015	39.9
25	26,828	2,721,021 - 2,747,829	38.0
26	44,154	2,747,834 - 2,791,432	37.4
27	80,647	2,791,881 - 2,869,494	39.4
28	161,418	2,869,526 - 3,027,146	38.9
29	162,938	3,027,172 - 3,189,672	39.3
30	846	3,190,537 - 3,189,692	38.2
31	68,355	3,190,561 - 3,258,915	36.8
32	15,000	3,258,920 - 3,273,919	38.8
33	23,640	3,273,939 - 3,297,578	38.2
34	2,471	3,300,052 - 3,297,582	47.4
35	434	3,300,057 - 3,300,490	49.0
36	2,612	3,303,106 - 3,300,495	50.2
37	34,268	3,303,109 - 3,337,376	38.5

38	3,280	3,340,658 - 3,337,379	37.4
39	23,182	3,340,663 - 3,363,844	38.6
40	43,327	3,407,175 - 3,363,849	40.8
41	19,667	3,426,851 - 3,407,185	38.0
42	26,632	3,426,856 - 3,453,482	39.4
43	31,755	3,453,484 - 3,485,231	39.0
44	20,074	3,485,236 - 3,505,224	39.3
45	2,515	3,505,229 - 3,507,743	44.9
46	4,570	3,512,148 - 3,507,747	32.5
47	2,127	3,514,277 - 3,512,151	32.5
48	42,710	3,514,339 - 3,557,045	37.4
49	20,743	3,577,794 - 3,557,052	39.2
50	843	3,577,814 - 3,578,656	42.8
51	37,693	3,578,827 - 3,616,519	38.6
52	8,025	3,624,546 - 3,616,522	38.7
53	33,930	3,624,551 - 3,658,480	39.3
54	2,482	3,658,484 - 3,660,965	37.4
55	2,421	3,663,401 - 3,660,981	40.5
56	36,310	3,663,418 - 3,699,727	38.9
57	11,144	3,699,743 - 3,710,886	38.9
58	3,960	3,710,898 - 3,714,857	38.1
59	7,654	3,722,515 - 3,714,862	37.0
60	36,420	3,722,532 - 3,758,644	38.5
61	10,140	3,768,788 - 3,758,649	38.1
62	803	3,769,613 - 3,768,811	43.2
63	821	3,769,630 - 3,770,450	43.1
64	3,515	3,770,577 - 3,774,091	36.4
65	1,126	3,774,113 - 3,775,238	35.8
66	2,099	3,775,242 - 3,777,340	41.4
67	3,887	3,781,237 - 3,777,351	37.2
68	2,960	3,784,886 - 3,781,927	33.0
69	5,241	3,790,338 - 3,785,098	38.7
70	1,305	3,790,340 - 3,791,644	39.5
71	18,158	3,791,675 - 3,809,386	37.5
72	3,606	3,813,022 - 3,809,417	36.2
73	7,705	3,813,027 - 3,820,731	36.9
74	1,263	3,820,770 - 3,822,010	44.1
75	1,610	3,823,198 - 3,824,807	48.9
76	962	3,826,505 - 3,825,544	44.9

*Relative to the *A. baumannii* AC30 chromosome.

Table S3. The presence of gaps of the *Acinetobacter* strains studied.

	AC30		11069/A	
Gap	Size (bp)	GC (%)	Size (bp)	GC (%)
1	1	100.0	1	100.0

2	1	0.0	1	0.0
3	2	50.0	1	0.0
4	2	50.0	1	100.0
5	2	50.0	2	0.0
6	2	50.0	2	50.0
7	2	50.0	2	0.0
8	3	66.7	3	0.0
9	3	33.3	3	66.7
10	3	66.7	3	33.3
11	3	0.0	4	25.0
12	3	33.3	6	33.3
13	3	33.3	17	64.7
14	3	33.3	60	41.7
15	3	33.3	108	33.3
16	4	0.0	119	35.3
17	4	25.0	129	53.5
18	4	25.0	136	52.2
19	4	25.0	148	20.3
20	4	50.0	158	44.3
21	4	0.0	170	32.4
22	4	0.0	194	30.4
23	4	0.0	199	34.7
24	4	50.0	224	43.8
25	4	50.0	230	43.9
26	4	25.0	241	41.9
27	4	25.0	245	48.2
28	4	25.0	251	41.8
29	4	25.0	273	37.7
30	4	0.0	284	39.4
31	4	25.0	305	32.2
32	4	75.0	377	41.1
33	4	25.0	428	46.3
34	4	25.0	437	39.8
35	5	20.0	446	30,7
36	6	0.0	449	41.9
37	7	42.9	451	39.2
38	8	50.0	465	39.6
39	9	11.1	466	30.5
40	9	44.4	487	42.3
41	10	50.0	490	37.5
42	11	18.2	492	43.1
43	11	54.5	498	41.7
44	12	25.0	503	41.4
45	15	53.3	523	43.0
46	15	26.7	528	53.4

47	16	25.0	596	40.9
48	16	25.0	647	37.6
49	16	37.5	736	42.0
50	19	42.1	781	42.3
51	19	57.9	826	38.4
52	19	26.3	936	38.5
53	19	36.8	962	39.2
54	21	38.1	994	47.2
55	22	36.4	997	43.3
56	23	34.8	1,021	42.5
57	24	54.2	1,165	41.0
58	25	28.0	1,490	37.6
59	30	36.7	1,607	35.0
60	30	33.3	1,766	38.6
61	31	45.2	1,798	42.0
62	38	42.1	1,932	51.8
63	61	26.2	1,990	40.6
64	97	64.9	2,419	42.8
65	126	35.7	2,735	36.7
66	170	43.5	3,412	41.6
67	211	41.7	3,630	43.7
68	448	41.5	3,963	47.9
69	633	41.4	4,428	46.9
70	689	41.9	4,620	39.8
71	736	47.4	11,256	42.4
72	1,139	35.5	15,800	39.5
73	1,187	38.2	21,402	34.2
74	8,034	38.5	37,788	41.1
75	62,520	38.3	103,465	38.5
76	73,834	37.6		

Table S4. The presence of mobile genetic elements including insertion sequences (ISs) identified on the gaps of the *Acinetobacter* strains studied.

**The Insertion Sequences**		
**IS Family**	**AC30**	**11069/A**
IS1-like	0	2
IS4-like element	2	14
IS5	0	1
IS6-like element IS26 family transposase	0	7
IS30	0	2
IS66	0	3
Transposase	0	10
Mobile element	0	3
In total	2	42

Table S5. Genomes of *Acinetobacter baumannii* ST195^{Ox} isolates used in this study.

Strain	Geographical Location	Size (Mb)	Year Isolation	Accession Number
11069/A	Czech Republic	3.81861	2018	PRJNA728954
AB07	India	4.24261	2011	CP006963
AC12	Malaysia	3.85523	2011	CP007549
AC30	Malaysia	3.92286	2011	CP007577
Ab-3	China	3.92776	2016	SVUL00000000
AB263	Saudi Arabia	3.97315	2012	LYNI00000000
ABE12_M	Morocco	3.90253	2015	FPEF00000000
AC_2355	Lebanon	4.02376	2016	MJBA00000000
CCF1	USA	3.94584	2015	LYZL00000000
2012046	China	3.80122	2012	NDXK00000000
T173	Thailand	3.93684	2010	JRTY00000000

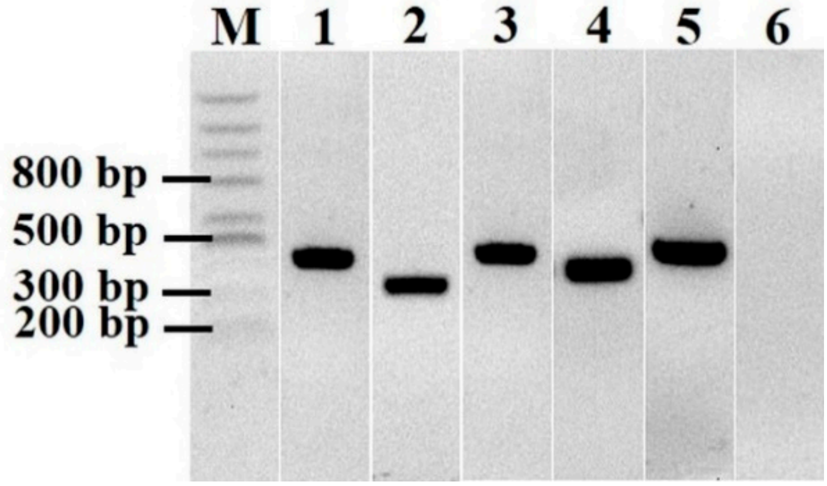

Figure S1. PCR products obtained by amplification of *β*-lactamase genes using specific primers in a clinical isolate 11069/A of *Acinetobacter baumannii*. Notes, lane 1, $bla_{\text{ADC-like}}$ gene (using the ADC-F1/R1 primers); lane 2, $bla_{\text{OXA-23}}$ gene (OXA(2)-F/R primers); lane 3, $bla_{\text{OXA-51-like}}$ gene (OXA(5)-F/R primers); lane 4, $bla_{\text{OXA-58-like}}$ gene (OXA(6)-F/R primers); lane 5, $bla_{\text{TEM-like}}$ gene; lane 6, negative control. M, molecular mass markers (200-1500 bp DNA ladder). The other 18 gene products ($bla_{\text{CARB-like}}$, $bla_{\text{CTX-M-like}}$, $bla_{\text{GES-like}}$, $bla_{\text{IMP-like}}$, $bla_{\text{KPC-like}}$, $bla_{\text{NDM-like}}$, $bla_{\text{OXA-2-like}}$, $bla_{\text{OXA-24-like}}$, $bla_{\text{OXA-48-like}}$, $bla_{\text{OXA-134-like}}$, $bla_{\text{OXA-143-like}}$, $bla_{\text{OXA-211-like}}$, $bla_{\text{OXA-213-like}}$, $bla_{\text{OXA-228-like}}$, $bla_{\text{PER-like}}$, $bla_{\text{SHV-like}}$, $bla_{\text{VEB-like}}$, and $bla_{\text{VIM-like}}$) were not generated with the DNA from this sample (data not shown).

life

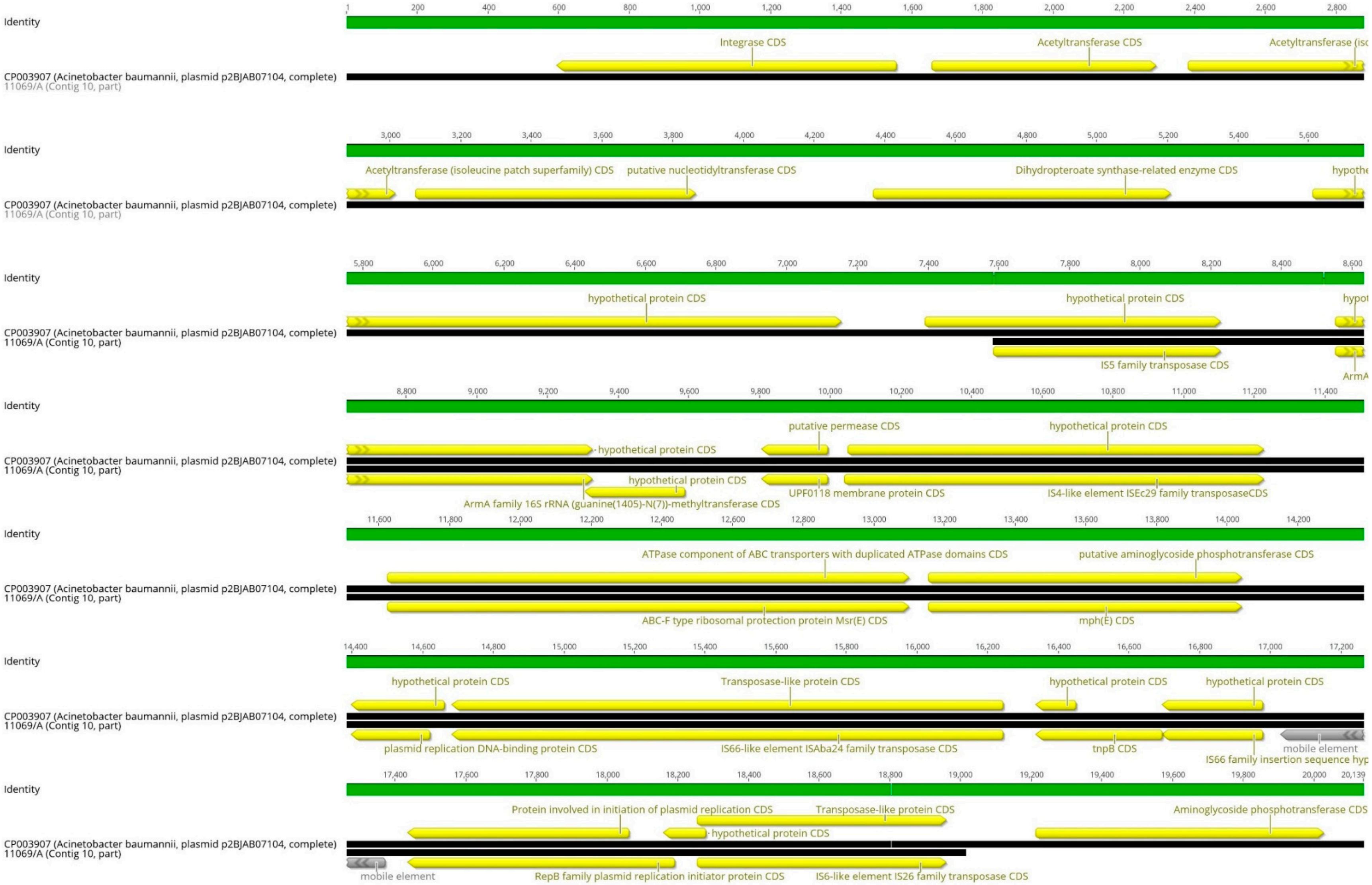

Figure S2. Linear comparison of the complete nucleotide sequence of plasmid p2BJAB07104 from *Acinetobacter baumannii* BJAB07104 with putative plasmid found in this study. The arrows indicate the positions and direction of transcription of the genes. Sequence comparison and map generation were performed using Geneious.

life

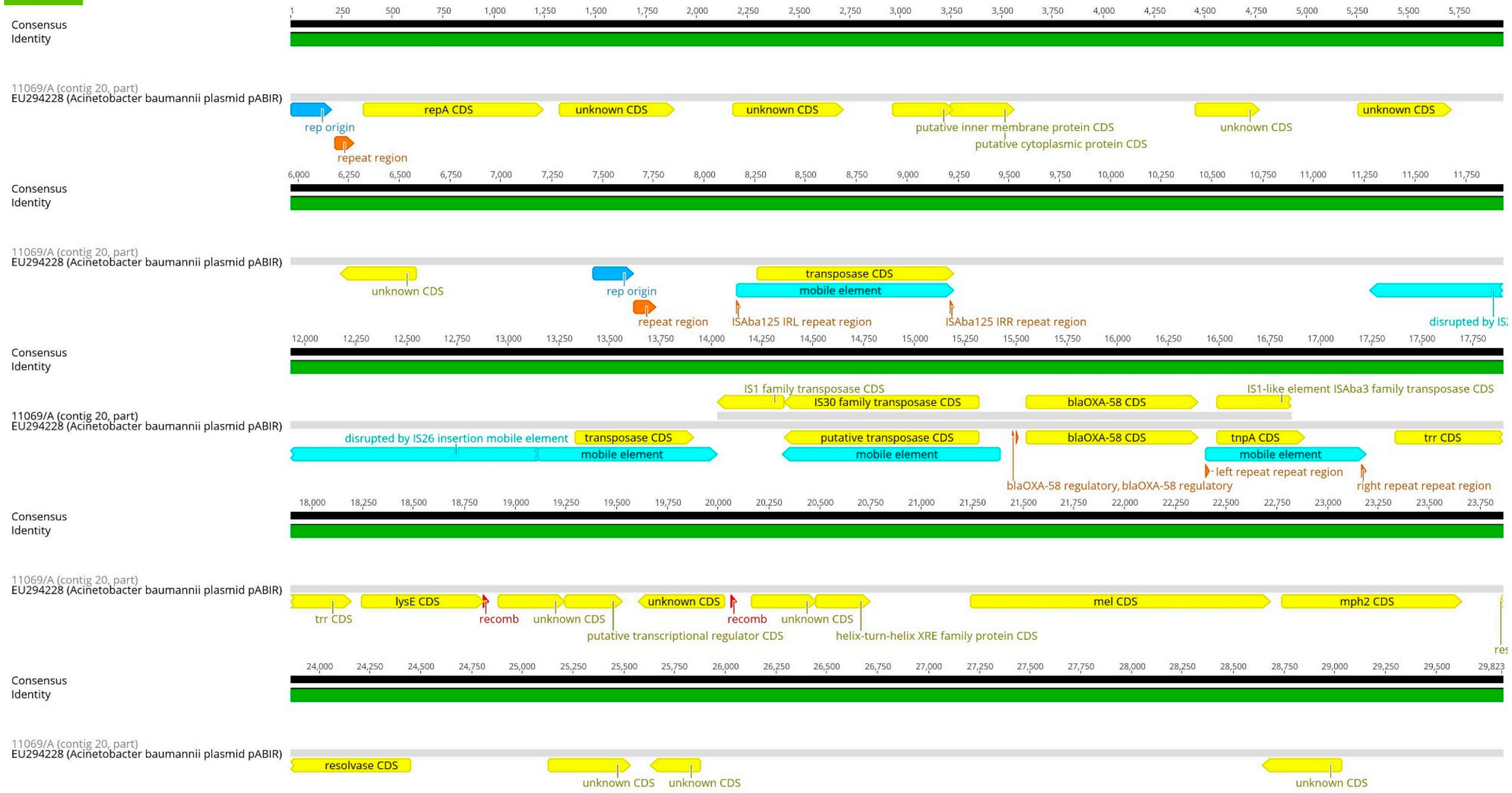

Figure S3. Linear comparison of the complete nucleotide sequence of plasmid pABIR from *Acinetobacter baumannii* with putative plasmid found in this study. The arrows indicate the positions and direction of transcription of the genes. Sequence comparison and map generation were performed using Geneious.

life

MDPI

References

1. Mlynarcik, P.; Dolejska, M.; Vagnerova, I.; Kutilova, I.; Kolar, M. Detection of clinically important beta-lactamases by using PCR. *FEMS Microbiol Lett* **2021**, *368*, doi:10.1093/femsle/fnab068.
2. Pagani, L.; Dell'Amico, E.; Migliavacca, R.; D'Andrea, M.M.; Giacobone, E.; Amicosante, G.; Romero, E.; Rossolini, G.M. Multiple CTX-M-Type extended-spectrum b-lactamases in nosocomial isolates of *Enterobacteriaceae* from a hospital in northern Italy. *Journal of Clinical Microbiology* **2003**, *41*, 4264–4269, doi:10.1128/Jcm.41.9.4264-4269.2003.
3. Kolar, M.; Htoutou Sedlakova, M.; Urbanek, K.; Mlynarcik, P.; Roderova, M.; Hricova, K.; Mezerova, K.; Kucova, P.; Zapletalova, J.; Fiserova, K.; et al. Implementation of Antibiotic Stewardship in a University Hospital Setting. *Antibiotics (Basel)* **2021**, *10*, doi:10.3390/antibiotics10010093.
4. Poirel, L.; Nordmann, P. Genetic structures at the origin of acquisition and expression of the carbapenem-hydrolyzing oxacillinase gene bla(OXA-58) in Acinetobacter baumannii. *Antimicrob Agents Ch* **2006**, *50*, 1442–1448, doi:10.1128/Aac.50.4.1442-1448.2006.
5. Mlynarcik, P.; Bardon, J.; Htoutou Sedlakova, M.; Prochazkova, P.; Kolar, M. Identification of novel OXA-134-like beta-lactamases in Acinetobacter lwoffii and Acinetobacter schindleri isolated from chicken litter. *Biomed Pap Med Fac Univ Palacky Olomouc Czech Repub* **2019**, *163*, 141–146, doi:10.5507/bp.2018.037.
6. Mlynarcik, P.; Roderova, M.; Kolar, M. Primer Evaluation for PCR and its Application for Detection of Carbapenemases in Enterobacteriaceae. *Jundishapur J Microbiol* **2016**, *9*, e29314, doi:10.5812/jjm.29314.
7. Mlynarcik, P.; Chalachanova, A.; Vagnerova, I.; Holy, O.; Zatloukalova, S.; Kolar, M. PCR Detection of Oxacillinases in Bacteria. *Microb Drug Resist* **2020**, doi:10.1089/mdr.2019.0330.
8. Lee, C.R.; Lee, J.H.; Park, M.; Park, K.S.; Bae, I.K.; Kim, Y.B.; Cha, C.J.; Jeong, B.C.; Lee, S.H. Biology of Acinetobacter baumannii: Pathogenesis, Antibiotic Resistance Mechanisms, and Prospective Treatment Options. *Front Cell Infect Microbiol* **2017**, *7*, 55, doi:10.3389/fcimb.2017.00055.
